# Residual Atherothrombotic Risk After Myocardial Infarction: Integrating Lipid, Inflammatory and Thrombotic Pathways

**DOI:** 10.3390/jcm15166358

**Published:** 2026-08-18

**Authors:** Alberto Sarti, Giorgio Sciaramenti, Giovanni Camaiti, Pierpaolo Cioci, Cristina Rizza, Renè Tezze, Ludovica Rita Vocale, Kristi Hoxha, Isabella Maccaferri, Francesco Paparazzo, Paolo Cimaglia, Andrea Erriquez, Rita Pavasini, Gianluca Campo

**Affiliations:** Cardiology Unit, Azienda Ospedaliero Universitaria di Ferrara, 44124 Ferrara, Italy; sarticchi@gmail.com (A.S.); giorgio.sciaramenti@gmail.com (G.S.); camaitigiovanni@gmail.com (G.C.); pierpaolocioci@gmail.com (P.C.); cristinarizza.94@gmail.com (C.R.); ludovica.vocale@gmail.com (L.R.V.); isabella.maccaferri@edu.unife.it (I.M.); francescopaparazzo1@gmail.com (F.P.); paolo.cimaglia88@gmail.com (P.C.); andrea.erriquez90@gmail.com (A.E.); pvsrti@unife.it (R.P.)

**Keywords:** myocardial infarction, residual atherothrombotic risk, secondary prevention, precision medicine

## Abstract

Despite major advances in reperfusion strategies and guideline-directed medical therapy, patients surviving myocardial infarction (MI) remain at substantial risk of recurrent cardiovascular events. Among the multiple determinants of post-MI cardiovascular risk, persistent atherothrombotic vulnerability remains a major therapeutic challenge despite guideline-directed secondary prevention. This review provides an integrated overview of residual atherothrombotic risk after MI, focusing on the complementary roles of lipid, inflammatory, and thrombotic pathways. Current evidence supports the use of biomarkers such as apolipoprotein B, lipoprotein(a), remnant cholesterol, triglyceride-rich lipoproteins, and high-sensitivity C-reactive protein to improve risk stratification beyond LDL-C. Landmark clinical trials have demonstrated that intensive lipid-lowering therapy, selected anti-inflammatory agents, and individualized antithrombotic strategies can further reduce recurrent cardiovascular events in appropriately selected patients. However, these pathogenic pathways rarely occur in isolation and frequently overlap, generating heterogeneous residual risk phenotypes. We propose a pragmatic multi-layered model in which lipid, inflammatory, and thrombotic mechanisms are viewed as interconnected biological processes rather than independent entities. This framework supports a personalized approach to secondary prevention based on comprehensive risk assessment, targeted therapeutic intensification, and longitudinal reassessment. Integrating these complementary domains may provide a framework for more individualized secondary prevention after MI, although its clinical utility requires prospective validation.

## 1. Introduction

Over the past decades, major advances in the management of myocardial infarction (MI), including timely reperfusion strategies and the widespread implementation of guideline-directed medical therapy (GDMT), have led to a substantial reduction in acute and long-term mortality [1]. Nevertheless, survivors of MI continue to experience a considerable burden of recurrent ischemic events, including recurrent myocardial infarction, ischemic stroke, and cardiovascular death, highlighting that optimal treatment does not completely eliminate future atherothrombotic risk [2].

This persistent vulnerability has led to the emergence of the concept of “residual atherothrombotic risk”, defined as the risk that remains despite adherence to evidence-based therapies and achievement of conventional therapeutic targets [3]. Importantly, post-MI residual cardiovascular risk is a broad concept encompassing multiple determinants, including ventricular dysfunction, arrhythmias, cardiorenal and metabolic comorbidities, lifestyle factors, participation in cardiac rehabilitation, and adherence to guideline-directed therapies. The present review specifically focuses on its residual atherothrombotic component, driven by lipid-related, inflammatory, and thrombotic pathways. Rather than representing a single entity, residual atherothrombotic risk reflects the coexistence of multiple biological pathways that contribute to atherothrombotic disease progression and recurrent events [3]. Consequently, patients with apparently similar clinical profiles may exhibit markedly different mechanisms underlying their ongoing susceptibility [3].

Growing evidence suggests that residual risk extends beyond low-density lipoprotein cholesterol and encompasses persistent dyslipidemia, chronic vascular inflammation, enhanced thrombotic propensity, and their complex interactions [4]. Recognizing these mechanisms is becoming increasingly important in the era of precision cardiovascular medicine, where individualized therapeutic strategies may provide additional benefit beyond standard care.

The aim of this review is to provide an integrated overview of the principal biological determinants of residual atherothrombotic risk after MI, focusing on lipid-related, inflammatory, and thrombotic mechanisms and available therapeutic approaches, while proposing a pragmatic framework for their clinical assessment and management.

**Figure 1:**
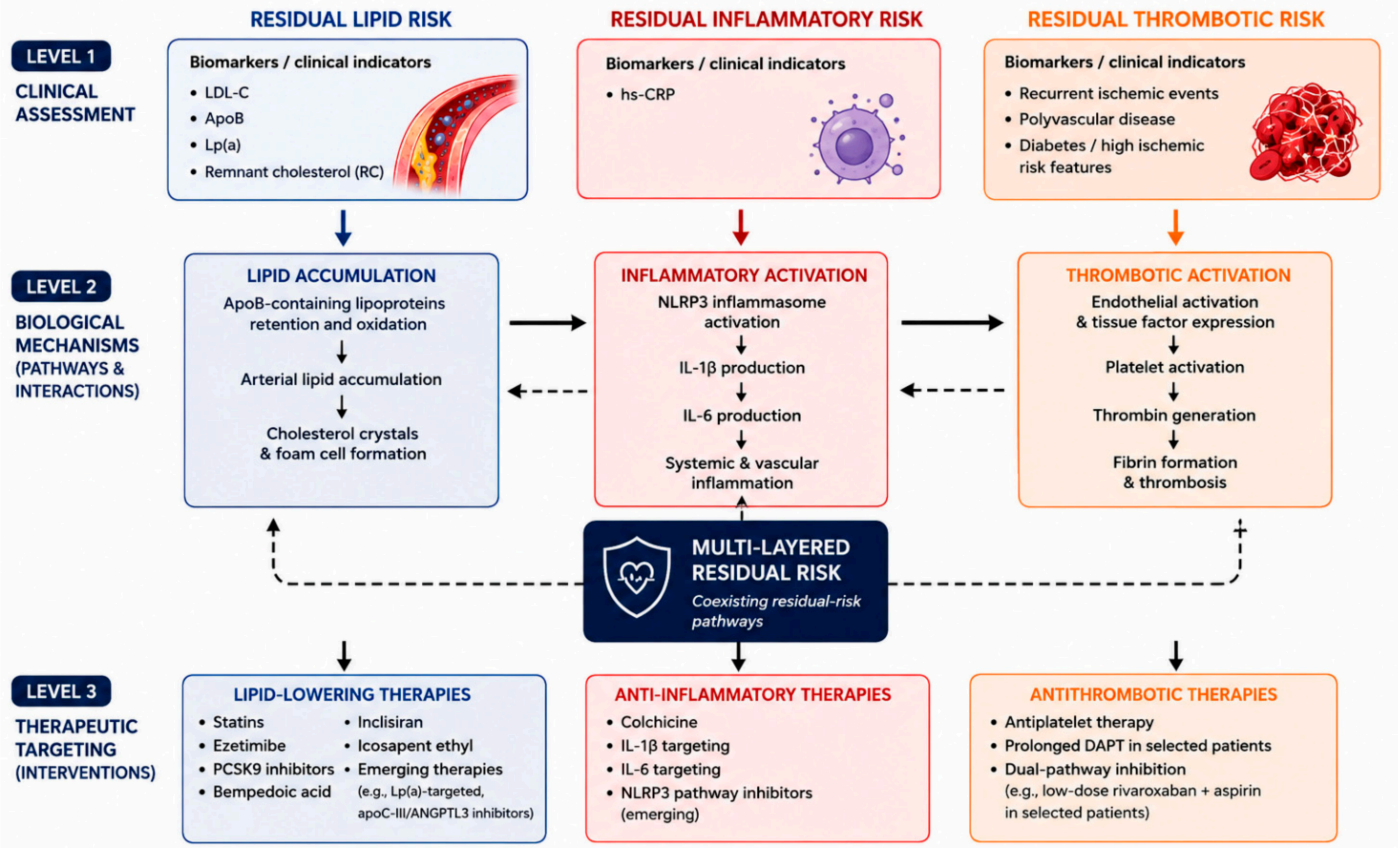
**Multi-layered model of residual atherothrombotic risk after myocardial infarction.** LDL-C: low-density lipoprotein cholesterol; Lp(a): lipoprotein(a); hs-CRP: high-sensitivity C-reactive protein; DAPT: dual antiplatelet therapy; RC: remnant cholesterol.

First, we will define the concept of residual risk and discuss its current classification. We will then examine the major components of residual lipid risk, inflammatory risk, and thrombotic risk, highlighting the evidence supporting targeted interventions in each domain. Particular attention will be paid to the frequent overlap between these residual-risk domains, as many patients simultaneously express multiple pathogenic mechanisms. Finally, we introduce the concept of ‘multi-layered atherothrombotic risk’, a model that views post-MI residual atherothrombotic risk as the result of interconnected lipid, inflammatory, and thrombotic processes rather than isolated abnormalities, supporting a more integrated and personalized approach to secondary prevention (Figure 1 and Table 1).

## 2. Methods

A targeted literature search was conducted in PubMed/MEDLINE to support this narrative review. Articles published in English from database inception to 30 June 2026 were considered. The search strategy included combinations of the following terms: “myocardial infarction”, “residual atherothrombotic risk”, “residual risk”, “secondary prevention”, “residual lipid risk”, “residual inflammatory risk”, “residual thrombotic risk”, “inflammation”, “platelet activation”, “thrombosis”, “atherothrombosis”, and “immunothrombosis”, together with terms related to established and emerging therapeutic strategies. Eligibility was restricted to articles published in English in peer-reviewed journals and addressing the mechanisms, assessment, or treatment of residual atherothrombotic risk after myocardial infarction. Particular emphasis was placed on landmark randomized clinical trials, observational studies, clinical guidelines, consensus documents, and emerging evidence relevant to residual lipid, inflammatory, and thrombotic risk. Ongoing clinical trials were also considered to provide an overview of future therapeutic strategies and unmet clinical needs. Studies were selected based on their clinical relevance, methodological robustness, and contribution to the understanding, assessment, or treatment of residual atherothrombotic risk. Given the narrative nature of the review, no formal systematic review or meta-analysis was performed.

## 3. Major Determinants of Residual Atherothrombotic Risk

### 3.1. Residual Lipid Risk

In the secondary prevention setting following an acute ischemic event, European guidelines recommend achieving low-density lipoprotein cholesterol (LDL-C) levels below 55 mg/dL in patients at very high cardiovascular risk [5,6]. Furthermore, the 2025 Focused Update of the ESC/EAS Guidelines for the Management of Dyslipidaemias recommends considering an LDL-C target below 40 mg/dL in a selected population of patients, including those with recurrent cardiovascular events despite optimized medical therapy or with polyvascular atherosclerotic disease [6].

Despite achieving guideline-recommended LDL-C targets, a substantial proportion of patients continue to experience major adverse cardiovascular events (MACE). This observation has reinforced the concept that residual atherothrombotic risk is a multidimensional phenomenon driven by overlapping lipid, inflammatory, and thrombotic pathways despite optimal guideline-directed therapy [5,6].

#### 3.1.1. Beyond LDL-C: Biomarkers of Residual Lipid Risk

Among the biomarkers most strongly associated with residual risk, non-high-density lipoprotein cholesterol (non-HDL-C) and apolipoprotein B (apoB) have emerged as key tools for cardiovascular risk stratification. Non-HDL-C reflects the cholesterol content of all apoB-containing lipoproteins, whereas apoB directly quantifies the total number of circulating atherogenic particles. Several studies have demonstrated that apoB is a more accurate marker of residual atherothrombotic risk than LDL-C in patients treated with statins, statin-ezetimibe combination therapy, or PCSK9 inhibitors, as well as in individuals with obesity, metabolic syndrome, type 2 diabetes mellitus, or insulin resistance [7,8,9].

Additional contributors to residual atherothrombotic risk include lipoprotein(a) [Lp(a)], triglycerides, and remnant cholesterol. Lp(a) consists of an LDL-like particle containing apolipoprotein B-100, covalently bound to apolipoprotein(a), which confers unique biological and pathogenic properties. Lp(a) is currently recognized as a genetically determined, independent, and causal risk factor for atherosclerotic cardiovascular disease and calcific aortic valve stenosis [10,11,12]. Moreover, circulating Lp(a) concentrations exhibit marked interindividual and interethnic variability, suggesting substantial differences in cardiovascular risk exposure across populations [11].

Remnant cholesterol refers to the cholesterol content carried by triglyceride-rich lipoproteins and their metabolic remnants. Following hydrolysis by lipoprotein lipase, chylomicrons and very-low-density lipoproteins (VLDL) are progressively converted into chylomicron remnants and VLDL/intermediate-density lipoprotein (IDL) remnants, particles characterized by a pronounced atherogenic potential and an enhanced ability to penetrate the arterial wall [13].

In parallel, a growing body of evidence from epidemiological, genetic, and Mendelian randomization studies has established a causal role for triglycerides in atherosclerosis and ischemic cardiovascular events [14,15]. These findings support the concept that triglyceride-rich lipoproteins represent an important therapeutic target complementary to LDL-C lowering.

Importantly, apoB, non-HDL-C, remnant cholesterol, and triglycerides are overlapping but distinct measures of residual lipid risk. apoB reflects the number of circulating atherogenic particles, whereas non-HDL-C reflects their cholesterol content. Remnant cholesterol specifically represents the cholesterol carried by triglyceride-rich lipoprotein and therefore represents a component of the non-HDL-C and apoB burden rather than an entirely separate lipoprotein fraction. At the same time, triglycerides reflect the circulating triglyceride concentration rather than particle number.

Consequently, discordance between these measures, particularly in patients with hypertriglyceridaemia or metabolic disturbances, may provide complementary information on the underlying atherogenic lipoprotein burden.

#### 3.1.2. Pharmacological Management of Residual Lipid Risk

From a therapeutic perspective, statins remain the cornerstone of lipid-lowering therapy through inhibition of 3-hydroxy-3-methylglutaryl-coenzyme A (HMG-CoA) reductase. The addition of ezetimibe, which inhibits intestinal cholesterol absorption by targeting Niemann-Pick C1-Like 1 (NPC1L1), enables further LDL-C reduction and is associated with a proportional decrease in cardiovascular event rates [16,17].

In patients who fail to achieve recommended lipid targets despite maximally tolerated statin therapy, PCSK9 inhibitors represent the next step in treatment intensification. Proprotein convertase subtilisin/kexin type 9 (PCSK9) promotes intracellular degradation of LDL receptors, thereby reducing hepatic clearance of circulating LDL particles. Inhibition of PCSK9 results in an approximately 60% reduction in LDL-C levels from baseline and a relative reduction of nearly 15% in major cardiovascular events, as demonstrated in the FOURIER and ODYSSEY OUTCOMES trials [18,19,20].

Beyond their effects on LDL-C, PCSK9 inhibitors significantly reduce circulating Lp(a) concentrations, likely through a combination of enhanced clearance and reduced production [21,22]. However, the frequently observed discordance between LDL-C and Lp(a) reductions suggests the involvement of additional biological mechanisms [23]. In a prespecified analysis of the FOURIER trial, an absolute reduction in Lp(a) of approximately 34 nmol/L was estimated to be necessary to achieve a 20% relative reduction in cardiovascular risk, particularly among patients with baseline Lp(a) concentrations exceeding 165 nmol/L [24].

Bempedoic acid represents an additional therapeutic option, particularly for patients who are unable to tolerate statins. By inhibiting ATP citrate lyase, an enzyme acting upstream of HMG-CoA reductase in the cholesterol biosynthetic pathway, bempedoic acid increases LDL receptor expression and enhances LDL particle clearance from the circulation [25]. Clinical studies have demonstrated LDL-C reductions exceeding 30% from baseline, together with a significant reduction in cardiovascular events among statin-intolerant individuals [26].

With regard to residual hypertriglyceridemia, icosapent ethyl (EPA) has shown a significant reduction in major cardiovascular events in statin-treated patients with established atherosclerotic cardiovascular disease or diabetes mellitus with additional cardiovascular risk factors, fasting triglyceride levels of 135–499 mg/dL, and LDL-C levels of 41–100 mg/dL. However, these findings should not be extrapolated to other omega-3 fatty acid preparations, as clinical trials evaluating other formulations have not consistently demonstrated cardiovascular benefit [27].

Among emerging therapeutic approaches, inclisiran, a small interfering RNA (siRNA), reduces hepatic PCSK9 synthesis through selective silencing of PCSK9 messenger RNA. In patients with atherosclerotic cardiovascular disease and persistently elevated LDL-C levels despite maximally tolerated lipid-lowering therapy, inclisiran has been shown to reduce LDL-C levels by approximately 50%; however, definitive evidence of cardiovascular outcome benefit remains pending [28].

#### 3.1.3. Emerging Therapeutic Targets for Residual Lipid Risk

In parallel, several innovative therapeutic strategies are currently under development. These include antisense oligonucleotides and siRNA-based therapies specifically targeting Lp(a) synthesis and metabolism, as well as inhibitors of cholesteryl ester transfer protein (CETP), a key mediator of cholesterol ester exchange among lipoprotein classes [28,29,30,31,32].

Additional emerging targets include apolipoprotein C-III (apoC-III) and angiopoietin-like protein 3 (ANGPTL3), both of which play central roles in triglyceride-rich lipoprotein metabolism. Interest in these pathways is supported by genetic and pharmacological evidence demonstrating that modulation of apoC-III and ANGPTL3 activity can substantially reduce exposure to atherogenic lipoproteins and mitigate residual atherothrombotic risk [33].

Finally, advances in gene-editing technologies are opening new therapeutic perspectives. Ongoing studies are investigating the possibility of achieving permanent suppression of hepatic PCSK9 expression through targeted DNA modification, with the aim of providing durable reductions in exposure to atherogenic lipoproteins and long-term cardiovascular risk reduction [34].

Taken together, the progressive intensification of lipid-lowering strategies has enabled the achievement of increasingly lower LDL-C levels and substantial reductions in cardiovascular event rates. Nevertheless, a significant burden of residual atherothrombotic risk persists even among patients who achieve recommended LDL-C targets. These observations highlight the limitations of an LDL-C-centered approach and underscore the need for a broader therapeutic strategy addressing additional atherogenic determinants, including apoB-containing lipoproteins, Lp(a), triglycerides, and remnant cholesterol. Targeting these pathways represents one of the major challenges and opportunities in contemporary cardiovascular prevention.

### 3.2. Residual Inflammatory Risk

Although intensive lipid-lowering therapy remains the cornerstone of secondary prevention, achieving LDL-C targets does not abolish cardiovascular risk. Persistent vascular inflammation represents a distinct and complementary mechanism underlying recurrent ischemic events, giving rise to the concept of residual inflammatory risk (RIR). Recognition of this inflammatory component has fundamentally expanded the pathophysiological framework of atherosclerotic disease and opened new therapeutic opportunities beyond cholesterol lowering. Residual inflammatory risk (RIR) refers to the persistence of vascular and systemic inflammatory activity despite optimal control of conventional cardiovascular risk factors, particularly LDL cholesterol. Increasing evidence indicates that inflammation is not merely a consequence of atherosclerosis but an active driver of plaque progression, destabilization, and recurrent ischemic events. Accordingly, inflammatory activation has emerged as an independent therapeutic target, complementary to lipid-lowering, in secondary prevention after myocardial infarction.

#### 3.2.1. Clinical Assessment of Residual Inflammatory Risk

High-sensitivity C-reactive protein (hs-CRP) is the most extensively validated biomarker of residual inflammatory risk in clinical practice. Produced predominantly by hepatocytes under IL-6 stimulation, hs-CRP reflects low-grade systemic inflammation and correlates with vascular inflammatory activity. Unlike conventional CRP assays, high-sensitivity testing detects subtle increases in circulating concentrations, making hs-CRP particularly suitable for cardiovascular risk stratification rather than for identifying acute inflammatory or infectious conditions.

Experimental and clinical evidence consistently supports the role of inflammation throughout the natural history of atherosclerosis, from plaque formation to plaque destabilization and thrombosis. Within this framework, hs-CRP has emerged as a robust surrogate of persistent inflammatory activation and one of the strongest predictors of recurrent cardiovascular events. A meta-analysis by Kandelouei et al. demonstrated that statin therapy consistently reduces hs-CRP concentrations across a broad spectrum of cardiovascular conditions, confirming that lipid-lowering treatment also exerts clinically relevant anti-inflammatory effects [35]. Nevertheless, a substantial proportion of patients continue to exhibit elevated hs-CRP despite optimal medical therapy, identifying a subgroup with persistent residual inflammatory risk [36].

The prognostic value of hs-CRP has been consistently demonstrated in both observational studies and randomized clinical trials. Among patients with previous myocardial infarction, persistent hs-CRP levels above 2 mg/L are associated with a significantly higher risk of major adverse cardiovascular events and all-cause mortality [36]. Furthermore, repeated hs-CRP measurements appear to provide greater prognostic information than a single determination, as transient elevations may reflect intercurrent conditions such as infection, trauma, surgery, or acute systemic illness rather than chronic vascular inflammation [37]. From a practical perspective, hs-CRP values < 1 mg/L are generally considered indicative of low inflammatory burden, whereas concentrations ≥ 2 mg/L identify patients at increased cardiovascular risk and are widely used to define residual inflammatory risk [38,39,40]. Conversely, hs-CRP levels > 10 mg/L should prompt evaluation for alternative inflammatory or infectious disorders before cardiovascular risk assessment [38,39,40].

Beyond its role as a prognostic biomarker, hs-CRP has progressively evolved into a therapeutic biomarker. The observation that cardiovascular risk may remain elevated despite optimal LDL-C control shifted attention from inflammation as a marker of disease to inflammation as a potential therapeutic target. Evidence from the REGARDS study demonstrated that patients with elevated hs-CRP experienced a higher incidence of coronary events, stroke, and cardiovascular mortality despite well-controlled LDL-C concentrations, providing one of the earliest clinical demonstrations of residual inflammatory risk [41]. This concept was subsequently reinforced by the PROVE-IT TIMI-22 sub-study, which showed that patients achieving hs-CRP levels < 2 mg/L after myocardial infarction had better clinical outcomes irrespective of LDL-C reduction, suggesting that inflammatory control provides prognostic information beyond lipid lowering [42,43]. Similarly, the JUPITER trial demonstrated that rosuvastatin significantly reduced major cardiovascular events in apparently healthy individuals with normal LDL-C but elevated hs-CRP, establishing inflammation as a complementary therapeutic target and highlighting that part of the cardiovascular benefit of statins extends beyond cholesterol lowering [44,45]. Although JUPITER was a primary prevention trial and therefore does not provide direct evidence in post-MI patients, it provided important proof-of-concept evidence supporting the prognostic relevance of inflammatory risk beyond LDL-C [44,45].

Together, these studies established inflammation as a therapeutic target complementary to lipid lowering rather than simply a marker of cardiovascular risk. Although the inflammatory response is essential for myocardial repair after ischemic injury, persistent low-grade inflammation promotes adverse ventricular remodeling and recurrent ischemic events [46,47]. More recent evidence confirms that elevated hs-CRP remains independently associated with mortality and major adverse cardiovascular events even after successful revascularization and optimal LDL-C control [48,49].

#### 3.2.2. Targeting Inflammation in Secondary Prevention

The recognition of inflammation as a therapeutic target stems from an improved understanding of the molecular pathways linking lipid accumulation to innate immune activation. Among these, the NLRP3 inflammasome has emerged as the central regulator of vascular inflammation. Cholesterol crystals activate NLRP3 within plaque macrophages, promoting the release of IL-1β and downstream IL-6 signaling, thereby sustaining vascular inflammation, plaque progression, and ultimately thrombotic complications [50,51,52,53,54,55].

Clinical validation of the inflammatory hypothesis came from the CANTOS trial, which provided the first evidence that selective inhibition of IL-1β reduces cardiovascular events independently of LDL-C lowering [56]. Despite proof-of-concept success, the modest absolute benefit, increased risk of fatal infection, and high treatment costs have prevented widespread clinical adoption.

Subsequent trials focused on more accessible anti-inflammatory strategies. In the post-MI setting, the COLCOT trial demonstrated that low-dose colchicine significantly reduced cardiovascular events after a recent myocardial infarction [57]. Complementary evidence from the LoDoCo2 trial, conducted in patients with chronic coronary disease rather than specifically in a post-MI population, further supported the cardiovascular benefit of long-term low-dose colchicine [58,59,60]. Conversely, the neutral results of CIRT demonstrated that not all anti-inflammatory approaches are equally effective, emphasizing that therapeutic benefit depends on selective inhibition of disease-relevant pathways rather than non-specific immunomodulation [61,62].

Based on these data, low-dose colchicine has been incorporated into contemporary guidelines as an adjunctive option for selected patients with persistent residual inflammatory risk, with the 2024 ESC chronic coronary syndrome guidelines strengthening its recommendation compared with previous documents [63,64].

Although colchicine is generally well tolerated, several considerations should be taken into account before initiating treatment:

- Gastrointestinal adverse events: As demonstrated in the systematic review, colchicine use is associated with gastrointestinal adverse events, including diarrhea, nausea, and vomiting, in approximately 10% of patients. These symptoms tend to resolve spontaneously over the following days; however, treatment discontinuation should be considered if gastrointestinal symptoms persist [65].
- Drug–drug interactions: Colchicine is a substrate of cytochrome P450 3A4 (CYP3A4) and P-glycoprotein (P-gp). Concomitant administration with potent CYP3A4 and/or P-gp inhibitors, such as clarithromycin, erythromycin, ketoconazole, cyclosporine, or verapamil, may substantially increase systemic colchicine exposure and consequently the risk of serious toxicity, including myopathy, rhabdomyolysis, and bone marrow suppression [66].
- Renal and hepatic impairment: Colchicine undergoes hepatic metabolism, and severe hepatic impairment may therefore result in drug accumulation and an increased risk of toxicity. Given that colchicine is also eliminated by the kidneys, its use should be avoided in patients with severe renal impairment, particularly in those with an estimated glomerular filtration rate (eGFR) below 15 mL/min [67].
- Infection and malignancy: Despite the demonstrated cardiovascular benefits of colchicine in patients with chronic coronary disease, the LoDoCo2 trial reported an increase in mortality in the colchicine group, primarily driven by fatal infections and malignancies. Although subsequent meta-analyses did not confirm a statistically significant increase in mortality, the use of colchicine should be avoided or carefully reconsidered in patients with an active severe infection or sepsis [68].

Overall, colchicine shows an acceptable safety profile at low doses, although gastrointestinal intolerance remains the most frequent adverse effect and concerns regarding non-cardiovascular mortality continue to be debated [69]. Post hoc analyses further suggest that earlier initiation after MI may maximize clinical benefit [61].

#### 3.2.3. Emerging Anti-Inflammatory Therapies

The success of CANTOS has driven increasing interest toward inflammatory pathways downstream of IL-1β, particularly interleukin-6 (IL-6), a cytokine produced by immune and vascular cells that plays a central role in atherogenesis and plaque progression [70,71,72]. Experimental evidence suggests that IL-6 signaling contributes to the maintenance of vascular inflammation, making it an attractive therapeutic target. This hypothesis was explored in the phase II RESCUE trial, in which ziltivekimab, a monoclonal antibody targeting IL-6, produced profound reductions in hs-CRP and other inflammatory biomarkers without affecting lipid levels, further supporting the concept that residual inflammatory risk is biologically distinct from residual lipid risk [73]. Building on these findings, the ongoing phase III ZEUS trial will determine whether IL-6 inhibition translates into a reduction in cardiovascular events in patients with chronic kidney disease and established atherosclerotic disease, while the ARTEMIS trial is specifically evaluating ziltivekimab in post-myocardial infarction patients [74,75]. Together, these studies will help determine the potential role of IL-6 inhibition as a targeted anti-inflammatory strategy for patients with persistent residual inflammatory risk.

Beyond cytokine inhibition, direct targeting of upstream inflammatory pathways is emerging as another therapeutic strategy. Experimental studies with selective NLRP3 inhibitors such as MCC950 have demonstrated reductions in infarct size, preservation of cardiac function, and attenuation of atherosclerosis in preclinical models [76,77]. Although hepatotoxicity has limited clinical development, these findings reinforce the concept that upstream inflammasome inhibition warrants further investigation as a potential anti-inflammatory therapeutic strategy.

### 3.3. Residual Thrombotic Risk

#### 3.3.1. Biological Basis of Residual Thrombotic Risk

Residual thrombotic risk reflects the persistence of prothrombotic mechanisms after myocardial infarction despite optimal secondary prevention. Although contemporary antithrombotic therapy and coronary revascularization have substantially reduced recurrent ischemic events, many patients remain exposed to ongoing platelet activation, thrombin generation, endothelial dysfunction, and vascular inflammation, all of which contribute to recurrent atherothrombotic complications [78,79,80].

Residual thrombotic risk should not be regarded solely as a local phenomenon related to the culprit lesion or coronary stent, but rather as the manifestation of a systemic atherosclerotic disease process involving multiple vascular territories [78].

Platelet hyperreactivity remains one of the key drivers of recurrent ischemic events after MI. Enhanced platelet activation may persist despite dual antiplatelet therapy (DAPT), particularly among patients with diabetes mellitus, chronic kidney disease, diffuse atherosclerotic disease, or previous recurrent ischemic events [80]. Persistent activation of platelet signaling pathways contributes not only to thrombus formation but also to vascular inflammation through platelet–leukocyte interactions, cytokine release, and endothelial activation [79,80]. These observations support the concept that platelets function as both thrombotic and inflammatory mediators within the atherosclerotic milieu. Beyond platelet activation, increasing evidence suggests that persistent activation of the coagulation cascade contributes substantially to residual thrombotic risk after MI [80]. Enhanced thrombin generation may persist long after the acute coronary event and promote recurrent thrombosis through fibrin formation, platelet activation, endothelial dysfunction, and inflammatory amplification [79,80]. This understanding has progressively shifted the therapeutic paradigm from isolated platelet inhibition toward strategies targeting both platelet aggregation and coagulation pathways, paving the way for the concept of dual pathway inhibition [80]. Inflammation and thrombosis are now recognized as deeply interconnected biological processes rather than independent pathways. Inflammatory cytokines promote endothelial dysfunction, tissue factor expression, platelet activation, and thrombin generation, whereas coagulation factors and activated platelets further amplify inflammatory signaling. This bidirectional interaction has been described as “immunothrombosis”, a physiological host-defense mechanism that becomes maladaptive in chronic cardiovascular disease. Dysregulated immunothrombosis contributes to thromboinflammation, promoting both microvascular and macrovascular thrombosis and ultimately increasing the risk of recurrent ischemic events [79]. Consistent with this concept, persistent inflammatory activation, reflected by elevated high-sensitivity C-reactive protein (hs-CRP) levels, has been associated with recurrent cardiovascular events despite optimal lipid-lowering therapy [60].

#### 3.3.2. Antithrombotic Strategies to Reduce Residual Risk

Long-term intensification of antithrombotic therapy after MI should be considered in selected patients in whom the expected reduction in recurrent ischemic events outweighs the associated bleeding risk. Beyond standard antithrombotic treatment following an acute coronary syndrome, two conceptually distinct approaches have been investigated for long-term secondary prevention: extended dual antiplatelet therapy (DAPT) and dual pathway inhibition (DPI) [1,68].

PEGASUS-TIMI 54 demonstrated that prolonged ticagrelor therapy reduces recurrent ischemic events at the expense of increased bleeding, supporting extended DAPT in carefully selected high-risk patients with prior MI [81]. Beyond the specifically post-MI setting, the COMPASS trial demonstrated the efficacy of dual pathway inhibition with low-dose rivaroxaban plus aspirin in patients with stable atherosclerotic vascular disease [82]. Although COMPASS was not restricted to post-MI patients, its findings provide complementary evidence supporting the concept of persistent thrombotic risk and intensified antithrombotic strategies in selected high-risk patients with established atherosclerotic disease [82].

The eligibility criteria and populations of these two trials are important when translating their findings into clinical practice. PEGASUS-TIMI 54 enrolled 21,162 clinically stable patients aged ≥50 years with a spontaneous MI 1–3 years before randomization and at least one additional high-risk feature: age ≥ 65 years, diabetes mellitus requiring pharmacological treatment, a second previous spontaneous MI, multivessel coronary artery disease, or chronic renal dysfunction [81]. Patients received ticagrelor 90 mg twice daily, ticagrelor 60 mg twice daily, or placebo on a background of low-dose aspirin. Both ticagrelor regimens reduced the composite endpoint of cardiovascular death, MI, or stroke compared with aspirin alone, although TIMI major bleeding was increased [81]. For long-term secondary prevention, ticagrelor 60 mg twice daily represents the clinically relevant regimen. Accordingly, the evidence from PEGASUS supports extended DAPT particularly in patients with previous MI and additional ischemic-risk features who have tolerated antiplatelet therapy without major bleeding and do not have a high bleeding risk [1,68,81].

COMPASS, in contrast, enrolled 27,395 patients with stable atherosclerotic vascular disease and randomized them to rivaroxaban 2.5 mg twice daily plus aspirin, rivaroxaban 5 mg twice daily alone, or aspirin alone [82]. The combination of low-dose rivaroxaban and aspirin reduced the composite endpoint of cardiovascular death, stroke, or MI compared with aspirin alone, while increasing major bleeding; importantly, fatal and intracranial bleeding were not significantly increased [82]. Unlike PEGASUS, previous MI was not an eligibility requirement: COMPASS included a broader population with stable coronary artery disease (CAD), peripheral artery disease (PAD), or both [82]. Moreover, patients requiring DAPT or therapeutic oral anticoagulation were excluded from COMPASS [82]. These differences are clinically relevant because COMPASS provides evidence for DPI in stable atherosclerotic vascular disease rather than specifically for a post-MI population.

Extended DAPT and DPI should therefore not be regarded as interchangeable strategies. PEGASUS evaluated prolonged platelet inhibition with aspirin and ticagrelor in patients selected specifically on the basis of previous MI and additional ischemic-risk features [81]. Conversely, COMPASS evaluated combined inhibition of platelet activation and factor Xa-mediated coagulation with aspirin and low-dose rivaroxaban in patients with stable CAD and/or PAD [82]. Thus, although both strategies aim to reduce residual thrombotic events, they differ in their pharmacological targets, evidence base, eligible populations, and clinical context. The choice of long-term antithrombotic intensification should consequently reflect the individual clinical setting and the balance between ischemic and bleeding risk rather than assuming therapeutic equivalence between the two approaches [1,68].

#### 3.3.3. Patient Selection and Risk Stratification

The decision to intensify antithrombotic therapy requires simultaneous assessment of ischemic and bleeding risk. Features associated with a higher risk of recurrent ischemic events include recurrent MI, diabetes mellitus, chronic kidney disease, multivessel coronary artery disease, peripheral artery disease, and extensive or polyvascular atherosclerosis [1,68,81,82]. These characteristics may support consideration of prolonged antithrombotic therapy, provided that bleeding risk remains acceptable.

Assessment of bleeding risk is therefore essential before extending or intensifying antithrombotic treatment. Relevant bleeding-risk factors include previous spontaneous or recurrent major bleeding, anemia, thrombocytopenia, advanced age or frailty, severe renal dysfunction, active malignancy, and previous intracranial hemorrhage [68,83,84,85]. Beyond risk stratification, potential contraindications to specific intensified antithrombotic regimens should be actively considered. Active clinically significant bleeding precludes antithrombotic intensification, while previous intracranial hemorrhage represents a major concern when prolonged potent antithrombotic therapy is considered. In addition, the COMPASS regimen should not be extrapolated to patients requiring therapeutic oral anticoagulation or concomitant DAPT, as these populations were excluded from the pivotal trial [82]. Renal function, concomitant medications, and other regimen-specific contraindications should also be reviewed before treatment initiation.

Several risk stratification tools have been developed to support individualized antithrombotic strategies. The DAPT score estimates the potential net benefit of extending DAPT beyond one year after percutaneous coronary intervention (PCI) by integrating both ischemic and bleeding predictors [83]. The PRECISE-DAPT score is primarily designed to estimate bleeding risk and may help identify patients in whom prolonged DAPT is less likely to provide a favorable risk–benefit balance [84]. The Academic Research Consortium for High Bleeding Risk (ARC-HBR) criteria provide a standardized definition of high bleeding risk in patients undergoing PCI, with at least one major or two minor criteria defining a high bleeding-risk profile [85]. More recently, the PRECISE-HBR score was developed and externally validated to further refine prediction of major bleeding after PCI [86]. These instruments provide complementary information but should support rather than replace individualized clinical assessment.

Importantly, ischemic and bleeding risks may change during follow-up. Development of anemia, worsening renal function, malignancy, recurrent bleeding, or a new indication for oral anticoagulation may substantially alter the risk–benefit balance of an initially appropriate extended antithrombotic regimen. Conversely, recurrent ischemic events or progression of atherosclerotic disease may indicate increasing thrombotic risk. Periodic reassessment is therefore appropriate in patients receiving long-term intensified antithrombotic therapy [1,68].

Platelet-function testing (PFT) and CYP2C19 genetic testing have also been investigated as tools to personalize antiplatelet treatment. High on-treatment platelet reactivity and CYP2C19 loss-of-function alleles can identify patients with reduced responsiveness to clopidogrel and have been associated with an increased risk of thrombotic events after PCI [87]. However, current evidence does not support the routine use of PFT or genetic testing in all patients, and their clinical utility for systematic residual thrombotic risk assessment remains limited [87]. These tests may instead be considered in selected clinical scenarios in which the result is expected to influence treatment decisions, including specific P2Y12 inhibitor escalation or de-escalation strategies or situations in which uncertainty regarding clopidogrel responsiveness has relevant therapeutic implications [87].

Overall, assessment of residual thrombotic risk should support selective rather than systematic intensification of antithrombotic therapy. Extended DAPT and DPI represent distinct evidence-based options for different clinical settings, and their use should be guided by the expected ischemic benefit, bleeding risk, and characteristics of the individual patient [68,81,82].

Future strategies will likely combine clinical scores with circulating biomarkers, advanced imaging, and precision medicine tools to better identify patients who derive the greatest net benefit from intensified antithrombotic therapy while minimizing bleeding complications (Table 2).

## 4. Toward an Integrated Model of Residual Atherothrombotic Risk

### 4.1. From Isolated Mechanisms to Biological Networks

Although residual lipid, inflammatory, and thrombotic risks are often described as distinct entities, accumulating evidence indicates that they represent highly interconnected biological processes rather than independent mechanisms. Lipid accumulation within the arterial wall promotes activation of the NLRP3 inflammasome through cholesterol crystal formation, triggering the release of pro-inflammatory cytokines such as IL-1β and IL-6. In turn, inflammation enhances endothelial dysfunction, platelet activation, tissue factor expression, and thrombin generation, creating a self-perpetuating cycle that accelerates atherosclerotic progression and recurrent cardiovascular events. Likewise, activated platelets amplify vascular inflammation through leukocyte recruitment and cytokine release, further reinforcing the concept of immunothrombosis as a central mechanism linking inflammation and coagulation.

### 4.2. A Multi-Layered Model of Residual Atherothrombotic Risk

This biological crosstalk suggests that residual atherothrombotic risk should not be interpreted through isolated pathways but rather as a multidimensional and dynamic process. Recognizing this biological interplay has important clinical implications. Rather than representing mutually exclusive entities, residual lipid, inflammatory, and thrombotic risks frequently coexist within the same patient. Accordingly, patient assessment should move beyond LDL-C alone and incorporate complementary domains, including apoB, Lp(a), hs-CRP, clinical history, atherosclerotic burden, and bleeding risk. Contemporary risk scores such as DAPT, PRECISE-DAPT, PRECISE-HBR, and ARC-HBR provide valuable information but remain focused on specific aspects of risk and do not fully capture the interaction between lipid metabolism, inflammation, and thrombosis.

Individual patients may exhibit abnormalities within one or more residual-risk domains. Persistent elevation of apoB or Lp(a) may indicate residual lipid risk, whereas persistent hs-CRP elevation despite optimal lipid control may identify an inflammatory component. Recurrent ischemic events, diffuse or polyvascular atherosclerotic disease, and other clinical or anatomical features associated with recurrent ischemic events may indicate increased ischemic/thrombotic risk. Importantly, these abnormalities should not be used to assign patients to mutually exclusive phenotypes. Instead, their coexistence should be interpreted as a multi-layered residual-risk profile, with the number and type of active domains informing subsequent clinical assessment and management.

### 4.3. Personalized Therapeutic Strategies

From a therapeutic perspective, management should address each clinically relevant and modifiable residual-risk domain according to the available evidence and specific treatment indications. Intensive lipid lowering with statins, ezetimibe, PCSK9 inhibitors, inclisiran, or emerging RNA-based therapies remains the cornerstone for residual lipid risk. Selected patients with persistent inflammatory risk may benefit from targeted anti-inflammatory strategies such as colchicine or, in the future, IL-6 inhibitors, whereas extended dual antiplatelet therapy or dual-pathway inhibition may be considered in appropriately selected patients with high residual ischemic/thrombotic risk and acceptable bleeding risk. When multiple residual-risk domains coexist, therapeutic strategies may need to address more than one pathway, while avoiding indiscriminate treatment intensification and carefully considering the overall benefit–risk balance. This integrated, multi-layered approach requires prospective validation.

### 4.4. A Practical Framework for Clinical Management

**Figure 2:**
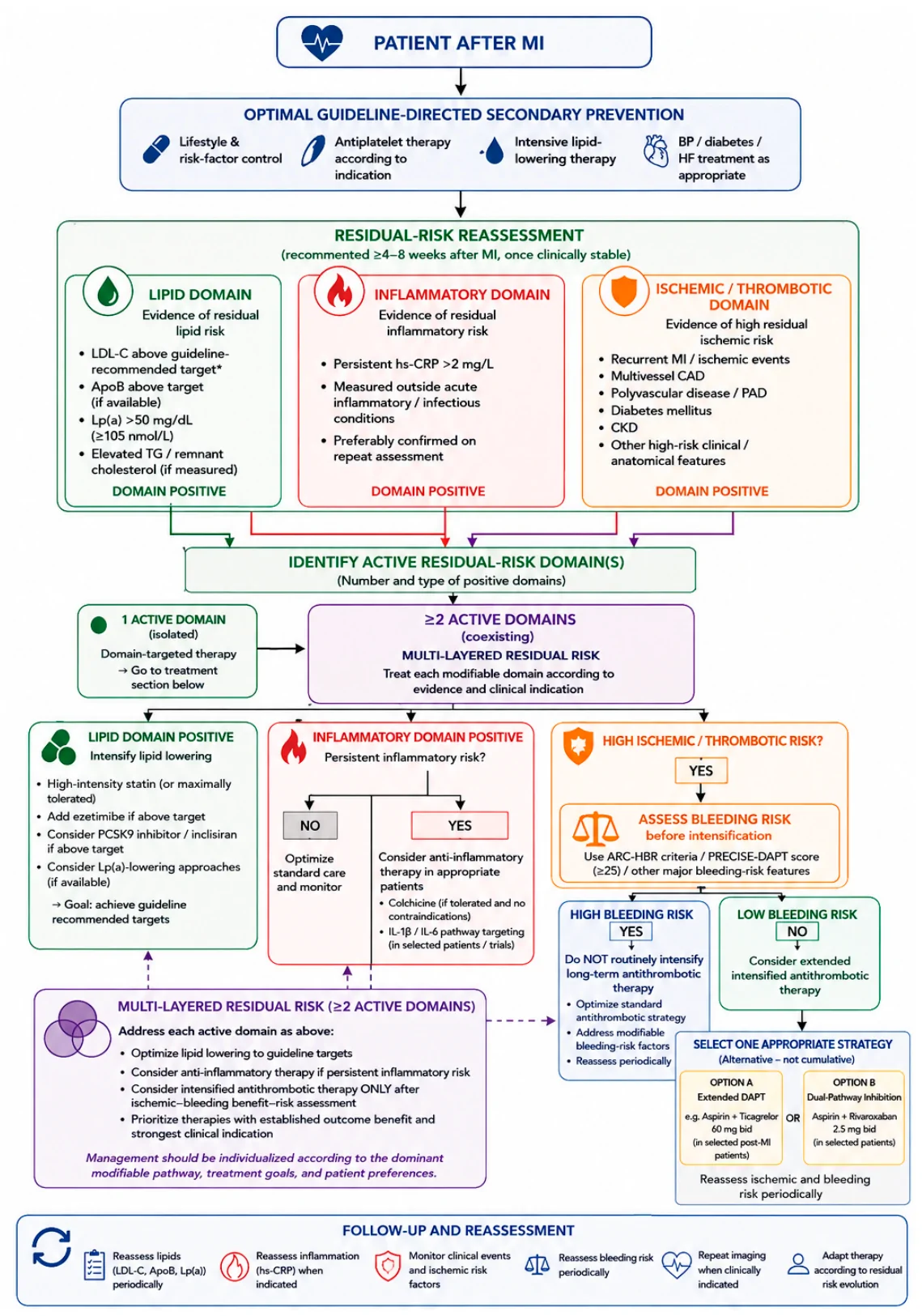
**Proposed clinical algorithm for the assessment and management of residual atherothrombotic risk after myocardial infarction.** MI: myocardial infarction; LDL: low-density lipoprotein; Lp(a): lipoprotein(a); hs-CRP: high-sensitivity C-reactive protein; DAPT: dual antiplatelet therapy; CKD: chronic kidney disease; PAD: peripheral artery disease; ARC-HBR: Academic Research Consortium for High Bleeding Risk. *: LDL-C target according to current guideline recommendations for patients after MI, taking into account the individual risk profile.

Based on current evidence, a pragmatic framework for the assessment and management of residual atherothrombotic risk after myocardial infarction may be proposed (Figure 2). The first step consists of ensuring optimal guideline-directed secondary prevention, followed by systematic reassessment of residual risk after the acute phase, when transient metabolic and inflammatory changes related to the index event have subsided. Assessment should encompass complementary domains, including conventional lipid parameters, apoB, Lp(a), hs-CRP, clinical and anatomical features associated with recurrent ischemic risk, atherosclerotic burden, and bleeding risk.

Rather than assigning patients to mutually exclusive predominant phenotypes, the proposed framework identifies the presence and number of active residual-risk domains. Lipid, inflammatory, and ischemic/thrombotic abnormalities may occur individually or coexist, with involvement of two or more domains representing a multi-layered residual-risk profile. Therapeutic management should address each clinically relevant and modifiable domain according to the available evidence and specific treatment indications. In patients with high residual ischemic/thrombotic risk, bleeding risk should serve as an explicit decision gate before considering intensified long-term antithrombotic therapy. When such intensification is appropriate, extended dual antiplatelet therapy and dual-pathway inhibition should be regarded as alternative, rather than cumulative, strategies selected according to individual clinical characteristics and the ischemic–bleeding balance. Periodic reassessment should account for changes in biomarkers, clinical status, ischemic risk, and bleeding risk over time.

### 4.5. Future Directions

Several challenges remain before this multidimensional framework can be translated into routine clinical practice. Most importantly, it has not been prospectively validated, and no randomized trial has evaluated whether a strategy based on the integrated assessment and targeted management of lipid, inflammatory, and thrombotic residual-risk domains improves clinical outcomes compared with contemporary guideline-directed secondary prevention. Furthermore, standardized criteria for defining individual residual-risk domains remain lacking, including optimal biomarker thresholds, timing and frequency of measurements after myocardial infarction, and criteria for distinguishing persistent abnormalities from transient changes related to the acute event. Evidence-based rules for interpreting discordant findings and prioritizing interventions when multiple domains coexist are also not established.

Additional limitations include the incomplete ability of current risk-prediction models to capture the biological interplay among lipid, inflammatory, thrombotic, and bleeding risk, as well as the cost and limited accessibility of several emerging therapies. Future studies should prospectively evaluate multidomain risk-assessment strategies, establish reproducible criteria for identifying clinically relevant residual-risk profiles, and determine whether targeting specific or coexisting pathways provides incremental clinical benefit. Integration of novel biomarkers, advanced imaging, and data-driven risk-prediction approaches may further refine patient selection; however, their clinical utility and impact on outcomes require prospective validation.

## 5. Conclusions

Secondary prevention after myocardial infarction has traditionally relied on intensive lipid-lowering and antithrombotic therapy. Contemporary evidence indicates that, although fundamental, these strategies do not completely eliminate recurrent atherothrombotic events. Residual atherothrombotic risk reflects the contribution and interplay of lipid-related, inflammatory, and thrombotic pathways, highlighting the limitations of considering individual risk mechanisms in isolation. A multidomain assessment integrating lipid parameters, inflammatory biomarkers, ischemic risk, and bleeding risk may provide a more comprehensive characterization of residual risk and help identify potentially modifiable pathways. Therapeutic decisions should remain grounded in established evidence and guideline recommendations, while emerging pathway-specific interventions require appropriate patient selection and further outcome validation. The proposed multidimensional framework may therefore provide a pragmatic basis for investigating more individualized approaches to secondary prevention after myocardial infarction, although its clinical utility requires prospective validation.

## Figures and Tables

**Table 1:** Characteristics of the three domains of residual atherothrombotic risk.

Characteristics	Lipid	Inflammatory	Thrombotic
Main mechanism	apoB-containing atherogenic lipoproteins	IL-1β/IL-6	Platelet activation
Main biomarkers	LDL-C, apoB-containing atherogenic lipoproteins, Lp(a), RC	hs-CRP	D-dimer, fibrinogen
Key mediators	LDL receptor pathway, PCSK9	NLRP3 inflammasome	Thrombin
Clinical phenotype	Hyperlipidemia	Persistent inflammation	Recurrent ischemia
Established therapies	Statins, Ezetimibe, PCSK9i, Bempedoic Acid, Inclisiran, Icosapent Ethyl	Colchicine	DAPT
Emerging therapies	Lp(a)-target RNA therapies, apoC-III/ANGPTL3-inhibition	IL-6/NLRP3-targeted therapies	Factor XI-targeted strategies

LDL-C: low-density lipoprotein cholesterol; Lp(a): lipoprotein(a); hs-CRP: high-sensitivity C-reactive protein; DAPT: dual antiplatelet therapy; RC: remnant cholesterol.

**Table 2:** Landmark trials targeting residual atherothrombotic risk.

Trial	Therapy Investigated	Main Findings	Clinical Implications
**RESIDUAL LIPID RISK**			
IMPROVE IT	Simvastatin + Ezetimibe	Additional LDL-C reduction with significant reduction in CV events	Established ezetimibe as first add-on therapy after statins
FOURIER	Evolocumab (PCSK9 inhibitor)	60% LDL-C reduction and reduction in MACE	Demonstrating benefit of intensive LDL-C reduction in secondary prevention
ODYSSEY OUTCOMES	Alirocumab (PCSK9 inhibitor)	Reduced recurrent CV events after recent ACS	Confirmed efficacy of PCSK9 inhibition in secondary prevention
CLEAR OUTCOMES	Bempedoic Acid	LDL-C reduction with lower event rates in statin-intolerant patients	Alternative lipid-lowering strategy for statin intolerance
REDUCE IT	Icosapent ethyl	Reduction in MACE in patients with hypertriglyceridemia	Highlighted triglyceride-rich lipoproteins as therapeutic target
**RESIDUAL INFLAMMATORY RISK**			
PROVE-IT (substudy)	Intensive statin therapy	Patients achieving hs-CRP < 2 mg/L had improved outcomes independently of LDL-C	Introduced concept of residual inflammatory risk
JUPITER	Rosuvastatin	Reduced CV events in patients with elevated hs-CRP despite normal LDL-C.	Demonstrated anti-inflammatory benefits of statins
CANTOS	Canakimumab	Reduced recurrent CV events without changes in LDL-C, but with an increased risk of serious infections	Demonstrated the potential benefit of targeted anti-inflammatory therapy, with infection risk representing an important limitation to its clinical use
COLCOT	Low-dose colchicine	Reduced MACE after recent MI, with generally good tolerability but potential gastrointestinal adverse effects and clinically relevant drug interactions	Supports colchicine for secondary prevention after recent MI, with patient selection and monitoring for tolerability and drug interactions
LODOCO2	Low-dose colchicine	Reduced CV events in CCS, with potential gastrointestinal intolerance and drug–drug interactions requiring consideration	Supports long-term anti-inflammatory therapy in selected patients with CCS, provided tolerability, renal/hepatic function, and potential interactions are considered
CIRT	Low-dose methotrexate	No reduction in inflammatory biomarkers or CV events	Demonstrated that non-specific immunomodulation is ineffective
RESCUE	Ziltivekimab (anti IL-6)	Marked hs-CRP reduction without lipid changes	Proof-of-concept for IL-6 inhibition
ZEUS	Ziltivekimab (anti IL-6)	Completed; results not yet published.	Will evaluate if IL-6 inhibition reduces MACE
ARTEMIS	Ziltivekimab (anti IL-6)	Ongoing trial	Will evaluate IL-6 inhibition after MI
**RESIDUAL THROMBOTIC RISK**			
PEGASUS-TIMI-54	Low dose of Ticagrelor + Aspirin	Reduced CV death, MI, and stroke, but with an increased risk of major bleeding	Supports prolonged DAPT in selected high-ischemic-risk patients, when the expected ischemic benefit outweighs bleeding risk
COMPASS	Low-dose of Rivaroxaban + Aspirin	Reduced CV death, MI, and stroke, with an increased risk of major bleeding	Established dual-pathway inhibition in selected patients with high ischemic risk, provided bleeding risk is acceptable

LDL-C: low-density lipoprotein cholesterol; CV: cardiovascular; MACE: major adverse cardiovascular events; ACS: acute coronary syndrome; hs-CRP: high-sensitivity C-reactive protein; MI: myocardial infarction; CCS: chronic coronary syndrome; DAPT: dual antiplatelet therapy.

## Data Availability

No new data were created or analyzed in this study. Data sharing is not applicable to this article.
